# Towards an Original Anti-ASFV Vaccine: Cellular Immunity Induced by Extracellular Vesicles Engineered with ASFV Proteins

**DOI:** 10.3390/vaccines14060514

**Published:** 2026-06-07

**Authors:** Francesco Manfredi, Flavia Ferrantelli, Chiara Chiozzini, Micaela Donnini, Patrizia Leone, Katherina Pugliese, Monica Cagiola, Cecilia Righi, Stefano Petrini, Monica Giammarioli, Francesco Feliziani, Maurizio Federico

**Affiliations:** 1 National Center for Global Health, Istituto Superiore di Sanità, Viale Regina Elena 299, 00161 Rome, Italy; francesco.manfredi@iss.it (F.M.); flavia.ferrantelli@iss.it (F.F.); chiara.chiozzini@iss.it (C.C.); micaela.donnini@iss.it (M.D.); patrizia.leone@iss.it (P.L.); katherina.pugliese@iss.it (K.P.); 2 National Reference Center for Swine Fever (CEREP), Istituto Zooprofilattico Sperimentale Umbria e Marche “Togo Rosati”, Via Gaetano Salvemini, 1, 06126 Perugia, Italy; m.cagiola@izsum.it (M.C.); c.righi@izsum.it (C.R.); s.petrini@izsum.it (S.P.); m.giammarioli@izsum.it (M.G.); f.feliziani@izsum.it (F.F.)

**Keywords:** African swine fever virus, extracellular vesicles, vaccines, HIV-1 Nef, cellular immunity

## Abstract

**Background/Objectives**: African Swine Fever (ASF) represents one of the most serious threats to animal health and global food security. The causative agent of ASF is the African swine fever virus (ASFV), a DNA virus belonging to the *Asfarviridae* family. Here, we describe ex vivo results for an original anti-ASFV vaccine approach based on the cellular immune response induced by extracellular vesicles (EVs) engineered to express four ASFV proteins. EV engineering was achieved by expressing a DNA vector encoding a biologically inactive HIV-1 Nef protein (Nef^mut^), which exhibits unusually high efficiency of incorporation into EVs, even when fused to foreign proteins. Previous studies have demonstrated that intramuscular injection of Nef^mut^-based vectors leads to the engineering of Evs, spontaneously released by muscle cells, and induction of antigen-specific CD8^+^ T cell immunity. **Methods**: We designed DNA vectors expressing the fusion products between Nef^mut^ and each of the four ASFV structural proteins p30, p54, pp62, and p72. Engineered EVs were molecularly characterized by Western blot and nanotrack analysis, and their potential immunogenicity was assessed by priming and cross-presentation assays. **Results**: We assessed that the four fusion proteins were successfully expressed in transfected mammalian cells, with the release of valuable amounts of engineered EVs. When immature swine dendritic cells were challenged with the engineered EVs and then co-cultivated with autologous peripheral blood lymphocytes in priming assays, lymphocyte subpopulations specifically reacting against each ASFV antigen were elicited, as detected by an IFN-γ ELISpot assay. In addition, we provide evidence that the Nef^mut^-based fusion products incorporated into the engineered EVs can be cross-presented by professional antigen-presenting cells, leading to cross-priming of autologous lymphocytes. **Conclusions**: These results represent the best premise to go forward with experiments examining immunogenicity and antiviral efficiency in pigs.

## 1. Introduction

African swine fever virus (ASFV) is the causative agent of the African swine fever (ASF). It is a DNA virus belonging to the *Asfarviridae* family, and it is known for its extraordinary resilience in the environment and the complexity of its genetic structure [1,2]. First identified in Kenya in the 1920s, ASFV recognized three major introductions thereafter: two into the Iberian Peninsula (in 1957 and 1960), leading to dissemination across parts of Europe, South America, and the Caribbean (eradicated by 1995); and a third introduction into Georgia in 2007, from which it has since spread across Europe, Asia, and the Caribbean.

Transmission occurs through direct contact among infected and susceptible animals, biological vectors such as soft ticks of the *Ornithodoros* genus, and contaminated material, including food and biological waste. In recent decades, ASF has emerged as a global health problem, with outbreaks reported in Asia, Europe, and Latin America. The rapid geographic spread of the virus has been facilitated by modern transportation and trade systems, as well as suboptimal farm management practices. These events have highlighted the need for an integrated strategy that combines epidemiological surveillance, improved biosecurity practices, and the development of innovative diagnostic and prophylactic tools.

ASF represents a major challenge for the global agri-food sector. Economic damage results not only from direct losses of pigs, but also from the cascading consequences that affect the entire production chain. Estimates of economic losses vary depending on the extent of the outbreak and the characteristics of the affected country. For example, in China, one of the world’s largest pork producers, ASF is estimated to have caused economic losses exceeding $100 billion between 2018 and 2020. In Europe, the impact has been particularly severe in Eastern European countries, where pig farming is a key component of the agricultural economy.

Despite significant efforts from the international scientific community, developing a safe and effective vaccine against ASFV remains a challenge [3]. This difficulty is attributable to a combination of factors, including the virus’s complexity, limited understanding of the immune mechanisms underlying viral pathogenesis, and the unique responses of infected hosts. Vaccine strategies against ASF have focused on inactivated viruses, live attenuated viruses, viral subunits, and vectored viral proteins. These approaches guarantee the production of high levels of anti-ASFV antibodies, which, however, even in the presence of cellular immunity, in most instances have been proven to be not adequate to counteract the infection with wild-type strains. In particular, reversion to infectious virus has been observed with live-attenuated vaccines [4], whereas poor protection was generated with subunit vaccines [5,6]. This evidence has prompted the shift in focus to a strong cellular immunity.

All cell types constitutively release nanovesicles collectively referred to as extracellular vesicles, EVs, which are key players in intercellular communication [7]. We developed a vaccine platform based on DNA vectors coding for antigens of interest fused at the C-terminus of a biologically inactive Human Immunodeficiency Virus (HIV)-Type 1 Nef protein (Nef^mut^) [8]. This protein mutant shows an unusually high efficiency of incorporation into EVs, which is maintained even when foreign polypeptides are fused to its C-terminus. Both N-terminal myristoylation and palmitoylation fasten Nef^mut^ to the luminal membrane leaflets, thus allowing its abundant uploading into EVs. Intramuscular injection of DNA vectors expressing Nef^mut^-related products leads to their incorporation in EVs constitutively released by muscle cells. These engineered EVs are internalized by professional antigen-presenting cells (APCs), and the incorporated products are processed to be loaded onto MHC complexes to induce strong antigen-specific immunity. Effectiveness and/or immunogenicity of this vaccine platform have been demonstrated with several viral products of various origins and sizes, including Human Papilloma Virus (HPV)16-E6 and -E7 [9], Ebola Virus VP24, VP40, and NP, Hepatitis C Virus NS3, West Nile Virus NS3, Crimean-Congo Hemorrhagic Fever NP [10], and SARS-CoV-2 N [11].

Given the relevance of cellular immunity to anti-ASFV vaccines, we planned to leverage the Nef^mut^ vaccine platform to design an original prophylactic approach against ASFV. To this end, four structural proteins having already documented intrinsic immunogenicity have been selected, namely p30, p54, pp62, and p72 [12]. Significantly, looking for T-cell epitopes with potential protective response, p30, pp62, and p72 were found to be the most potent inducer of IFN-γ production in lymphocytes from ASFV-immunized pigs [13], and epitopes from both pp62 and p72 proteins induced the strongest protective immunity [3,14].

The results from both in vitro and ex vivo immunologic assays aimed at establishing the potential immunogenicity of the four engineered EV types are reported here.

## 2. Materials and Methods

### 2.1. DNA Constructs

The pTarget-Nef^mut^ vector was already described [10]. To obtain the Nef^mut^-based fusion constructs, genotype II ASFV p30, p54, pp62, and p72 open reading frames (ORFs) were inserted in Apa I/Not I sites of the previously described intermediate DNA vector referred to as pTarget/Nef^mut^ fusion [10]. In this vector, the complete Nef^mut^ ORF deprived of its stop codon was followed by a sequence coding for a GPGP linker including a unique *Apa* I restriction site. The ASFV ORFs were inserted in their 5′ to 3′ orientation at the *Apa* I site of the GPGP linker, and at the immediately downstream Not I site. In this way, the ASFV ORFs were fused in frame with the Nef^mut^ ORF. Stop codons at the 3′ end of ASFV-related ORFs were preceded by sequences coding for a DYKDDDK epitope tag (flag-tag). The N-terminal 52 amino acids of the ASFV p54 protein (out of the 184 total amino acids) were not included in the fusion product in view of a strong transmembrane localization tendency of this domain [15], which can interfere with the correct interaction of Nef^mut^ with the cell membrane, possibly reducing its incorporation efficiency in EVs. DNA sequences were optimized for expression in pig cells through GenSmart™ Codon Optimization software from Genescript (https://www.genscript.com/tools/gensmart-codon-optimization, accessed on 12 May 2026, Piscataway, NJ, USA). All vectors were synthesized by OfficinaeBio (Venice, Italy).

### 2.2. Production and Characterization of Engineered EVs

EVs were isolated from transiently transfected human embryonic kidney (HEK) 293T cells (ATCC, CRL-11268), which were grown in DMEM (Gibco, Thermo Fisher, Waltham, MA, USA) plus 10% heat-inactivated fetal calf serum (FCS, Gibco, Thermo Fisher Scientific). Transfections were performed by the Lipofectamine 2000 (Invitrogen, Thermo Fisher Scientific)-based method. After 24 h, transfected cell cultures were washed and reseeded in medium containing EV-deprived FCS. From 48 to 72 h after transfection, Supernatants were harvested, and EVs were isolated through differential centrifugations [16] by centrifuging supernatants at 500× *g* for 10 min, and then at 10,000× *g* for 30 min. Supernatants were harvested, filtered with 0.22 µm pore size filters, and ultracentrifuged at 70,000× *g* for 1 h. Pelleted vesicles were washed in 1× PBS and ultracentrifuged again at 70,000× *g* for 1 h. Finally, pellets containing EVs were resuspended at 1:100 of the initial volume.

### 2.3. NTA of Nanovesicles

Purified EVs diluted in 1× PBS underwent nanoparticle tracking analysis (NTA) using a Nanosight NS300 with the NTA software v3.00 (Malvern Panalytical Ltd., Malvern, UK) through a 488 nm laser.

### 2.4. Western Blot Analysis

Transfected cells were washed twice with PBS (pH 7.4) and then lysed with 1× SDS-PAGE sample buffer. Samples were run in 10% sodium dodecyl sulfate-polyacrylamide gel electrophoresis (SDS-PAGE), and transferred on a 0.45 µM pore size nitrocellulose membrane (Amersham, Buckinghamshire, UK) by electroblotting carried out with a Bio-Rad Trans-Blot apparatus. EVs were lysed and analyzed in the same way. Membranes were incubated with 5% non-fat dry milk in PBS containing 0.1% Triton X-100 for 1 h at room temperature. Afterwards, the membranes were challenged overnight at 4 °C with the antibodies of interest in PBS containing 0.1% Triton X-100. In detail, 1:1000-diluted anti-Nef monoclonal antibody (mAb, clone 3E6, Invitrogen), sheep anti-Nef antiserum ARP 444 (MHRC, London, UK), 1:500-diluted anti-β-actin AC-74 mAb from Sigma, and 1:500-diluted anti-Alix H-270 polyclonal Abs from Santa Cruz (Dallas, TX, USA) were used. After incubation with HRP-specific substrate, the signals were detected by a Chemi-Doc apparatus, Bio-Rad, running the Image Lab software version 6.1 (Bio-Rad, Hercules, CA, USA).

### 2.5. Priming Assay

Cells were isolated from the peripheral blood mononuclear cells (PBMCs) of domestic pigs by the Ficoll-Paque method [17]. Monocyte and lymphocyte sub-populations were isolated after incubation with human anti-CD14 microbeads (Miltenyi, Bergisch Gladbach, Germany) and immunomagnetic separation following the manufacturer’s recommendations. CD14^+^ cells were seeded in 24/48 well plates at 2 × 10^6^ cells/mL in RPMI 10% of EV-deprived FCS supplemented with 50 μM β-mercaptoethanol in the presence of 50 ng/mL of porcine IL-4 (R&D System, Minneapolis, MN, USA) and 20 ng/mL of porcine GM-CSF (R&D System). After 6–7 days, i.e., the time needed to induce monocytes to differentiate towards immature dendritic cells (iDCs), a first cycle of treatment with about 100 particles/cell of the engineered EVs was carried out by spinoculation, i.e., plate centrifugation for 30 min at 800× *g* at room temperature. Afterwards, iDCs were induced to maturation by 1 µg/mL of LPS, and then autologous peripheral blood lymphocytes (PBLs) were added in a 1:10 cell ratio. A week later, the challenge procedure was repeated on freshly differentiated iDCs, which were then co-cultivated with the cells recovered from the first cell cycle. After an additional week, cells were recovered and counted for downstream IFN-γ EliSpot assays.

### 2.6. Cross-Presentation Assay

A total of 4 × 10^4^ cells of a human HLA-B7 B-lymphoblastoid cell line (B-LCL) were challenged by spinoculation as described above with about 10^7^ particles of the different EV preparations, and, after 3 h of incubation, were co-cultured at a 1:5 ratio with a Nef-specific, HLA-B7 restricted CD8^+^ T-cell clone [18] in the context of an IFN-γ EliSpot assay.

### 2.7. IFN-γ EliSpot Analysis

From 0.5 to 2.5 × 10^5^ live cells were seeded in replicate microwells (Millipore, Burlington, MA, USA) coated the day before with 1:100 diluted anti-pig IFN-γ mAb (clone P2G10, BD Pharmingen, Allschwil, Switzerland) in RPMI 1640, 10% FCS, and 50 μM β-mercaptoethanol. Cell cultures were carried out for 24 h in the presence of 1 μg/mL each of overlapping 15-mers specific either for Nef, or for each of the four ASFV proteins, or the same concentration of PTE (Principal CTL Epitopes) Nef-specific peptides (NIH HIV Reagent Program) [19]. ASFV pools of peptides were obtained by >70% pure preparations (BioFab, Rome, Italy) of 15-mers with 10 amino acid overlapping sequences. As a negative control, 1 μg/mL each of overlapping 15-mers specific for the SARS-CoV-2 S1 protein (BEI Resources Repository) was used. To check for cell responsiveness, 10 ng/mL of phorbol 12-myristate 13-acetate (PMA, Sigma, St. Louis, MO, USA) plus 500 ng/mL of ionomycin (Sigma) were added to cell cultures. The readout of the cross-presentation assay was carried out in EliSpot plate microwells coated with the 1-D1K anti-human IFN-γ antibody (Mabtech, Nacka Strand, Sweden). In this case, 5 μg/mL of the HLA-B7-restricted TPGPGVRYPL Nef peptide [14] was added as a positive control. In all instances, the cells were discarded after 24 h, and the plates were washed and incubated for 2 h at room temperature with either biotinylated pig anti-IFN-γ antibody (clone P2C11, BD) or biotinylated human anti-IFN-γ antibody (clone 7-B6-1, Mabtech), both at a concentration of 1 μg/mL. Wells were then washed and treated for 1 h at room temperature with 1:1000 diluted streptavidin-ALP (Mabtech). Finally, 100 μL of SigmaFast BCIP/NBT was added to each well to develop spots. Spot-forming units (SFUs) were counted by an AELVIS EliSpot reader (Hannover, Germany).

### 2.8. Statistical Analysis

When appropriate, data are presented as mean ± standard error of the mean (SEM), or as individual values and their mean. Statistical analyses were conducted with GraphPad Prism 9, using the Kruskal–Wallis’s test, followed by Dunn’s post-test for multiple comparisons.

## 3. Results

### 3.1. The Fusion Products Based on the Four ASFV Proteins Are Expressed in Mammalian Cells and Incorporated into EVs

**Figure 1:**
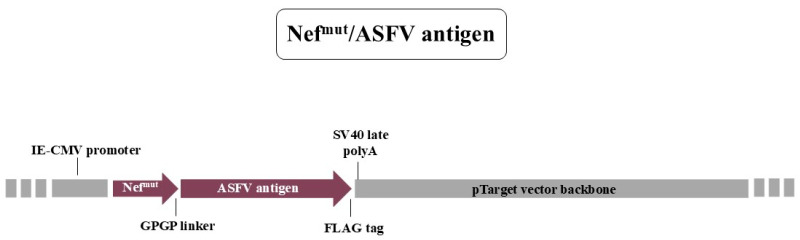
Map of the pTarget-based vectors expressing Nef^mut^-based fusion proteins. Shown are the positions of the vector IE-CMV promoter, the GPGP linker, the Flag-tag, as well as the vector SV40 poly-A signal.

**Figure 2:**
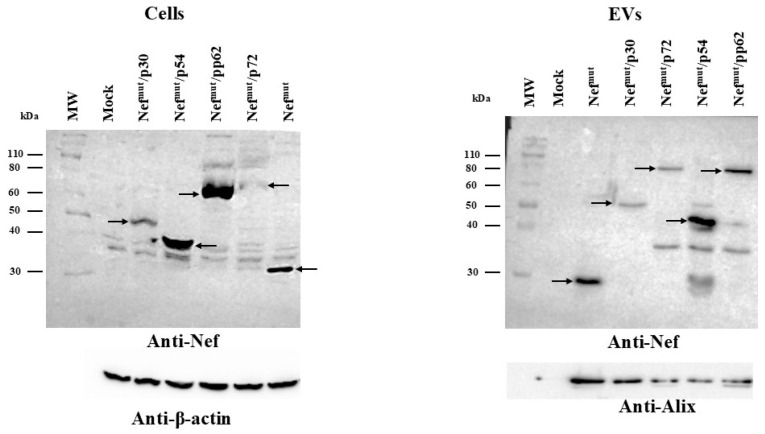
Detection of Nef^mut^-based fusion products in transfected cells and EVs. Left panel: Western blot analysis of 30 µg of lysates from HEK293T cells transfected with DNA vectors expressing Nef^mut^ either alone or fused with either p30, p54, pp62, or p72 ASFV ORFs. On the right panel, equal volumes of buffer where concentrated EVs were resuspended after differential centrifugations of the respective supernatants were analyzed. As controls, both cell lysates and EVs from mock-transfected cells were included. An anti-Nef monoclonal Ab was used to detect Nef^mut^-based products. β-actin and Alix were identified as markers in cell lysates and EVs, respectively. Arrows indicate relevant signals. Molecular markers are given in kilodaltons (kDa). The results are representative of three independent experiments.

DNA constructs coding for the products of fusion between Nef^mut^ and the four ASFV ORFs (Figure 1) were assayed for expression in mammalian cells. To this aim, HEK293T cells were transfected with each DNA construct, and the expression of the fusion products was checked by anti-Nef Western blot analysis (Figure 2 and Supplementary Figure S1). The four constructs appeared to be expressed in transfected cells at levels comparable to those induced by the DNA expressing Nef^mut^ alone, except the Nef^mu^/p72 fusion product, whose low, still detectable expression level was confirmed by replicate experiments (Supplementary Figure S2). On the other hand, the Western blot analysis of EVs isolated from the supernatants of transfected cells demonstrated the apparently correct incorporation of all fusion products, which generated signals of intensities comparable to those from EVs incorporating Nef^mut^ alone.

**Figure 3:**
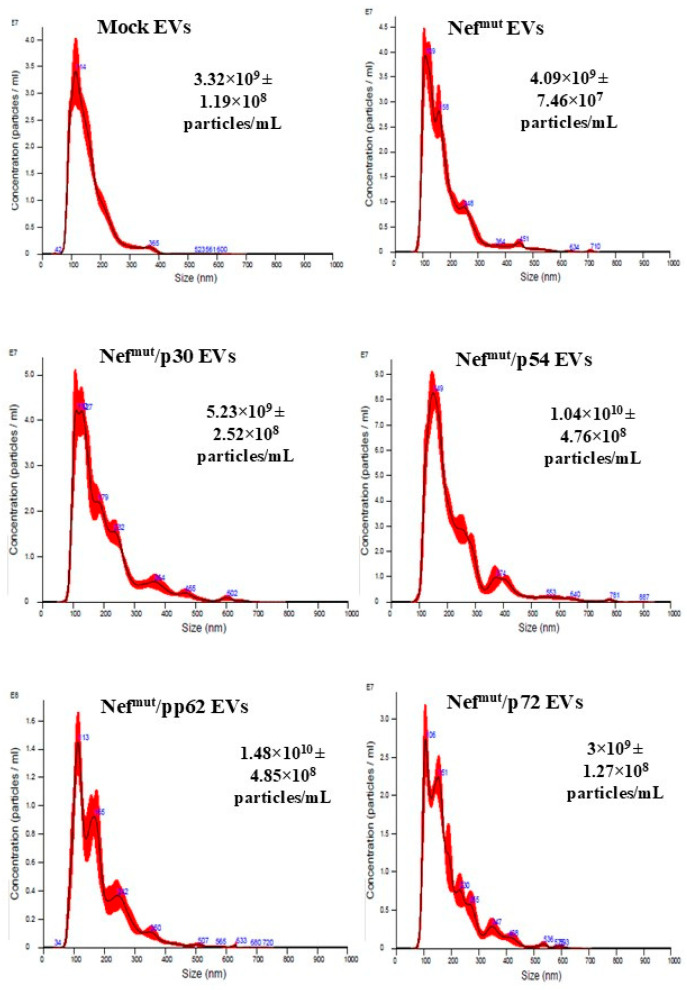
NTA of EVs isolated from the supernatants of HEK 293T transfected cells. EVs from cells transfected with either Nef^mut^ or Nef^mut^ fused with each of the four ASFV proteins were isolated by differential centrifugation and resuspended in equal volumes of buffer. As a control, EVs from mock-transfected cells were analyzed. Preparations were then diluted 20 times and analyzed 5 times with a Nanosight NS300 device. Size distribution (in blue), concentration measurements s.e. (in red), with mean values (in black), as well as average concentration of EVs ± s.e. are reported. The results are representative of two independent experiments.

The nanotrack analysis of the EV preparations (Figure 3) revealed no major quantitative differences among the six preparations considered. Notably, the nanotrack profile of all engineered EVs presented a double peak within the 200-nanometer interval, i.e., the filtration exclusion size before differential ultracentrifugation. This evidence suggests the presence of EVs of different sizes, i.e., EVs void and incorporating high amounts of Nef^mut^-derived products. Minor peaks of greater size likely reflected the presence of post-centrifugation aggregates.

From these data, we concluded that the four DNA constructs expressed fusion proteins with a stability adequate to forward them into EVs spontaneously produced by transfected cells.

### 3.2. Lymphocytes Are Primed to React Against the ASFV-Derived Peptides After Co-Cultivation with Autologous DCs Challenged with Engineered EVs

**Figure 4:**
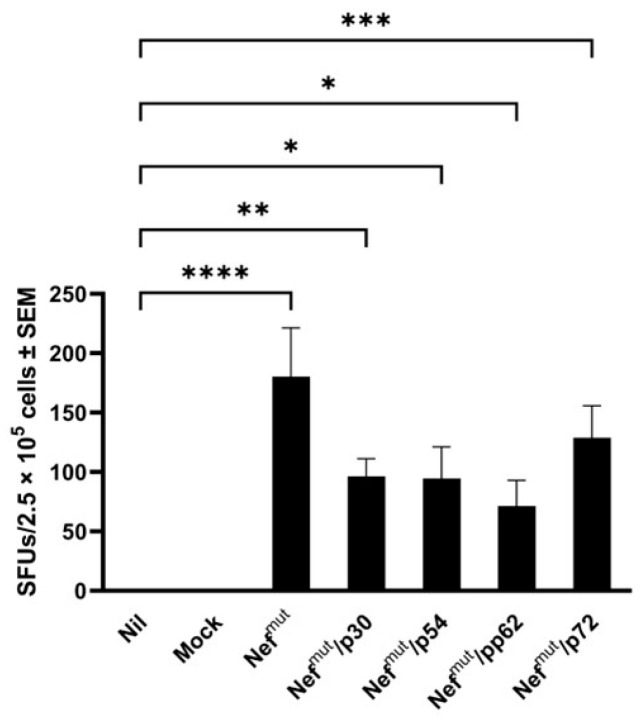
ASFV peptide-specific lymphocyte activation in DC-PBLs co-cultures after the priming assays, as detected by IFN-γ EliSpot analysis. From two to six IFN-γ EliSpot microwells were seeded with 0.5–2.5 × 10^5^ cells and incubated for 24 h with or without either specific or unrelated pools of overlapping 15-mers at a final concentration of 1 µg/mL each. Shown are the numbers of SFUs/2.5 × 10^5^ cells calculated as mean values after subtraction of mean spot numbers measured in wells with cells treated with unspecific peptides. Nil: cells recovered after co-cultivation with unchallenged DCs. Mock: cells recovered after co-cultivation with DCs challenged with EVs isolated from cells transfected with the void DNA vector. The results are from three independent experiments carried out with cells from different pigs. Reported are mean values ± standard errors of the mean. * *p* ≤ 0.05, ** *p* ≤ 0.01, *** *p* ≤ 0.001, **** *p* ≤ 0.0001.

The possibility of exploiting the four ASFV-based DNA constructs as a vaccine strictly relies on their ability to induce antigen-specific lymphocytes. This has been assayed through priming experiments carried out with PBMCs isolated from domestic pigs. CD14^+^ cells isolated from peripheral blood were differentiated into iDCs by the treatment with both porcine IL-4 and GM-CSF. After 6–7 days, 5 × 10^5^ iDCs were challenged in duplicate wells with about 5 × 10^7^ EVs from the different preparations. Thereafter, iDCs were matured with LPS and co-cultivated with 5 × 10^6^ lymphocytes. After two cycles of challenge and co-cultivation, the cells were harvested, counted, and tested in an IFN-γ EliSpot assay (Figure 4 and Supplementary Figure S3) using pools of overlapping 15-mers. The results indicated that, in all instances, statistically significant antigen-specific lymphocyte responses were elicited, with the highest detected in co-cultures including DCs treated with Nef^mut^ EVs. The frequencies of ASFV-specific lymphocytes were similar across treatments.

These results supported the idea that the challenge of porcine professional APCs with ASFV-based engineered EVs can lead to the priming of antigen-specific lymphocytes.

### 3.3. The Fusion Products Incorporated into Engineered EVs Can Undergo Cross-Presentation in Professional APCs

**Figure 5:**
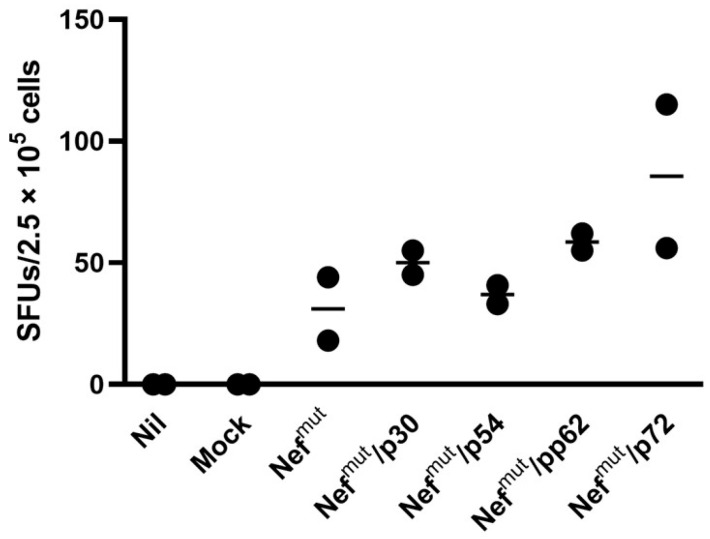
Cross-presentation in B-LCLs challenged with EVs engineered with ASFV products. HLA-B7 B-LCLs were challenged with equal amounts of Nef^mut^-based EVs. As control, the cells were treated with the same amounts of mock EVs or left untreated (Nil). B-LCLs were then put in co-culture overnight with HLA-B7-matched Nef-specific CD8^+^ T cells in an IFN-γ EliSpot setting. Shown are the means of SFUs/2.5 × 10^5^ CD8^+^ T cells calculated from two independent experiments.

Several lines of experimental evidence indicate that the Nef^mut^-based vaccine platform is highly effective at inducing antigen-specific CD8^+^ T lymphocytes [8]. To assess whether this feature can be reproduced with the ASFV-based DNA constructs, we performed cross-presentation experiments with human APCs. In detail, MHC Class I-B7 BLCLs were challenged with equal amounts of each Nef^mut^-based EV type and then co-cultivated with a B7-restricted anti-Nef CD8^+^ T cell clone in a 1:5 cell ratio in the context of an IFN-γ EliSpot assay. The actual cross-presentation of the molecules incorporated into engineered EVs was demonstrated by the evidence that the number of spots detected after the co-culture of CD8^+^ T cells with BLCLs challenged with the different EV preparations largely overcame those detected in control conditions, i.e., BLCLs treated with either vehicle (Nil) or EVs isolated from cells transfected with the void DNA vector (“mock” EVs) (Figure 5 and Supplementary Figure S4).

This result suggests that the ASFV-based fusion molecules incorporated into engineered EVs can undergo cross-presentation similarly to that occurring with Nef^mut^ alone.

### 3.4. Cross-Priming of Nef^mut^-Based Molecules Incorporated into Engineered EVs as Detected with PTE Peptides

**Figure 6:**
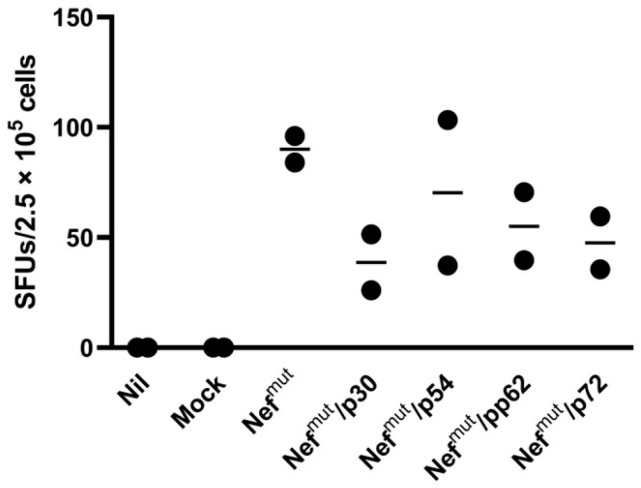
Cell activation induced by PTE-Nef peptides in DC-PBLs co-cultures after the priming assays. IFN-γ EliSpot microwells were seeded in replicate with 0.5–2.5 × 10^5^ cells and incubated for 24 h with or without either PTE-Nef or pools of unrelated peptides at a final concentration of 1 µg/mL each. Shown are the numbers of SFUs/2.5 × 10^5^ cells calculated as mean values after subtraction of the mean spot numbers detected in wells with cells treated with unspecific peptides. Nil: cells recovered after co-cultivation with unchallenged DCs. Mock: cells recovered after co-cultivation with DCs challenged with EVs isolated from cells transfected with the void DNA vector. The results are from two independent experiments carried out with cells from different pigs.

An efficient cross-presentation of the immunogenic molecules represents a prerequisite for the generation of an effective CD8^+^ T cell immune response. Next, we checked the possible effects of the cross-presentation of EV-associated Nef^mut^-related molecules in terms of cross-priming of porcine lymphocytes. To this end, priming experiments with porcine PBMCs were replicated, and the IFN-γ EliSpot readout assay was performed using Nef-specific PTE peptides, i.e., a pool of peptides designed to activate CD8^+^ T cells. Consistent with that observed in cross-presentation experiments, the results of the IFN-γ EliSpot assay (Figure 6) indicated that the treatment of iDCs with each engineered EV cross-primed a subpopulation of Nef-specific CD8^+^ T lymphocytes.

Together, these data are in line with the idea that each ASFV-based EV type can prime and cross-prime antigen-specific lymphocytes. This evidence can be considered a relevant issue from the perspective of testing this vaccine strategy in pigs.

## 4. Discussion

Ethical issues impose that, especially in the case of large animals, every new in vivo experimental approach should be justified by solid results obtained by in vitro/ex vivo preliminary assays. Accordingly, before translating the EV-based anti-ASFV vaccine strategy we propose into pigs, the immunologic potentialities of the candidate vaccines have been checked by laboratory tests.

Wide experimental evidence demonstrated that even a strong antibody response against ASFV proteins is not sufficient to protect the animals against infection [5,20]. This may relate to the virus transmission among monocyte/macrophages (i.e., the cell type primarily infected by ASFV) through apoptotic bodies, a mechanism that can shield the virus from antibody attack [21]. Moreover, anti-ASFV antibodies can facilitate the infection of alveolar macrophages through the ADE (antibody-dependent enhancement) mechanism [22], as demonstrated in infected pig also [23]. On the other hand, the protection observed in pigs vaccinated with live-attenuated ASFV strains was abolished after depletion of CD8^+^ T lymphocytes [24,25]. Hence, even if the immunologic correlate of protection against ASFV infection remains unknown, it is widely accepted that the cell-mediated adaptive immune response can play a key role in resistance to virus infection.

ASFV is a DNA virus with a genome of up to 193 kilobase pairs expressing more than 160 proteins. Clearly, the choice of viral protein candidates for a new vaccine formulation is expected to be based on previously established immunogenic features. This was the case of the four structural ASFV proteins we included in the Nef^mut^-based vaccine platform. In detail, ASFV p30 is a very early structural protein involved in viral particle internalization. Besides its strong humoral immunogenicity [26], a promiscuous cytotoxic T-cell epitope within highly conserved ASFV sequences has been identified [27]. Additional T-cell epitopes binding SLA-I, II, and III have been identified through computational analysis [28]. ASFV p54 is a 25-kilodalton protein containing a transmembrane domain at its N-terminal moiety. This protein is essential for the recruitment and transformation of intracellular membranes into viral envelope structures [15]. A T-cell epitope mapping at the 60–68 amino acid position has been identified [29]; thus, it occurred in a region not affected by the N-terminal 54 amino acid deletion necessary for the fusion with Nef^mut^. Furthermore, four additional potential T-cell epitopes presented in SLA-I complex have been identified [30]. pp62 is a polyprotein cleaved upon infection by the pS273R viral protease to form the mature virion proteins p8, p15, and p35, which are necessary for the viral core morphogenesis [31]. As many as seven pp62 highly conserved cytotoxic T-cell epitopes have been identified, three of which bind to at least two largely distributed SLAs in domestic pigs [27]. More recently, in silico analysis has identified four additional highly conserved, promiscuous pp62 CD8^+^ T cell epitopes [32]. Finally, ASFV p72 is the major viral capsid protein forming the viral icosahedron upon trimerization [33]. A study identified six highly conserved cytotoxic T-cell epitopes [27], and five more p72-related T-cell epitopes were described in additional investigations [34,35].

In sum, the previous identification of cytotoxic T-cell epitopes renders the four ASFV proteins we selected credible vaccine candidates in the context of the Nef^mut^-based vaccine platform, considering the already demonstrated ability of foreign proteins incorporated into EVs by the fusion with Nef^mut^ to elicit cytotoxic CD8^+^ T cell immunity [8]. In addition, still unidentified T cell epitopes could functionally contribute to the overall adaptive cell immune response induced by ASFV-based engineered EVs.

Notably, we already demonstrated that the effective cross-priming observed in ex vivo cell cultures challenged with engineered Nef^mut^-based EVs associated with a strong immunogenicity in vivo, which, in turn, is coupled with both antiviral and anticancer activity in animals. In detail, K18 transgenic mice vaccinated with DNA expressing the Nef^mut^/SARS-CoV-2 N fusion protein were protected from the infection with SARS-CoV-2 [11]. Moreover, the intramuscular injection of a DNA expressing Nef^mut^ fused with the tumor-associated antigen HER2/neu inhibited the spontaneous tumor cell growth in 129SvNeuT transgenic mice [36]. In this scenario, the results reported here represent the best premise to proceed toward studies of immunogenicity and efficacy in pigs.

The present study shows some limitations: first, engineered EVs have been produced in human cells and tested in pig PBMCs for both priming and cross-priming assays. It is known that EVs deliver macromolecules of cells from which they are generated [37,38]. Some background activity could have been generated by the delivery of EV-associated human molecules to pigs’ APCs, as well as by the cell internalization of proteins from the EV-deprived FCS. However, the reliability of our results is demonstrated by the levels of lymphocyte activation detected in the conditions where iDCs were treated with engineered EVs, which were both significantly and reproducibly higher than those observed in control conditions. Second, only a Nef-specific, HLA-B7-restricted epitope has been considered in the cross-presentation experiments. The unavailability of SLA-characterized CD8^+^ T cell clones specific to ASFV antigens did not allow us to extend the analysis to pig-specific epitopes. Nevertheless, in the same way that cross-presentation of Nef^mut^-associated products detected in vitro with human cells translated to mice [8,10,18], we assume that the here described cross-presentation data can translate to pigs. Finally, additional analysis, including intracellular cytokine staining, CD107a [39], and trogocytosis [40] assays, could have provided a more precise characterization of cytotoxic lymphocytes recovered after priming assays. However, the results we obtained with the use of PTE peptides support the idea that the population of lymphocytes activated after treatment of APCs with the ASFV-based EVs readily included cytotoxic, antigen-specific CD8^+^ T cells.

## 5. Conclusions

Cumulative data on pre-clinical models render the Nef^mut^-based vaccine platform a strong candidate to combat infectious agents refractory to antibody-mediated neutralization. Concerning ASFV, both simplicity and flexibility of the Nef^mut^-based vaccine platform (e.g., in terms of inclusion of most conserved sequences) serve to overcome two of the major pitfalls of the most recent anti-ASFV vaccines based on live-attenuated viral strains, i.e., the reversion to infectious virus, and the lack of protection against different ASFV genotypes.

Ongoing immunogenicity/efficacy experiments in domestic pigs will establish how the potential immunogenicity of engineered EVs may translate into protection against ASFV infection. Notably, based on in vivo studies carried out with both HPV-16 and SARS-CoV-2 proteins [8,9], the overall anti-ASFV cellular immune response induced by Nef^mut^-based EVs would be optimized by co-delivery of the most immunogenic DNA vectors.

## Data Availability

The data presented in this study are available on request from the corresponding author.

## Supplementary Materials

The following supporting information can be downloaded at: https://www.mdpi.com/article/10.3390/vaccines14060514/s1, Figure S1. Western blot analysis of transfected cells and EVs isolated from respective supernatants: raw data. Figure S2. Nef^mut^/p72 detection in replicate Western blot analysis of cells transfected with DNA expressing ASFV-based fusion proteins. Figure S3. IFN-γ EliSpot analysis after priming assay. Raw data. Figure S4. Cross-presentation assay. Raw data.

## Abbreviations

The following abbreviations are used in this manuscript:

APCs	Antigen-presenting cells
ASFV	African Swine fever virus
BLCL	B-cell lymphoblastoid cell line
DCs	Dendritic cells
EVs	Extracellular vesicles
HLA	Human leukocyte antigen
MHC	Major histocompatibility complex
PBMCs	Peripheral blood mononuclear cells
PTE	Principal CTL epitopes
SLA	Swine leukocyte antigen
