# Supplementary material for: Towards an Original Anti-ASFV Vaccine: Cellular Immunity Induced by Extracellular Vesicles Engineered with ASFV Proteins
Source: Vaccines (Basel). 2026 Jun 7;14(6):514. doi: 10.3390/vaccines14060514 (PMC13308295; doi:10.3390/vaccines14060514)
Supplement: Supplementary file 1 [file vaccines-14-00514-s001.zip › vaccines-4345701-supplementary.pdf]

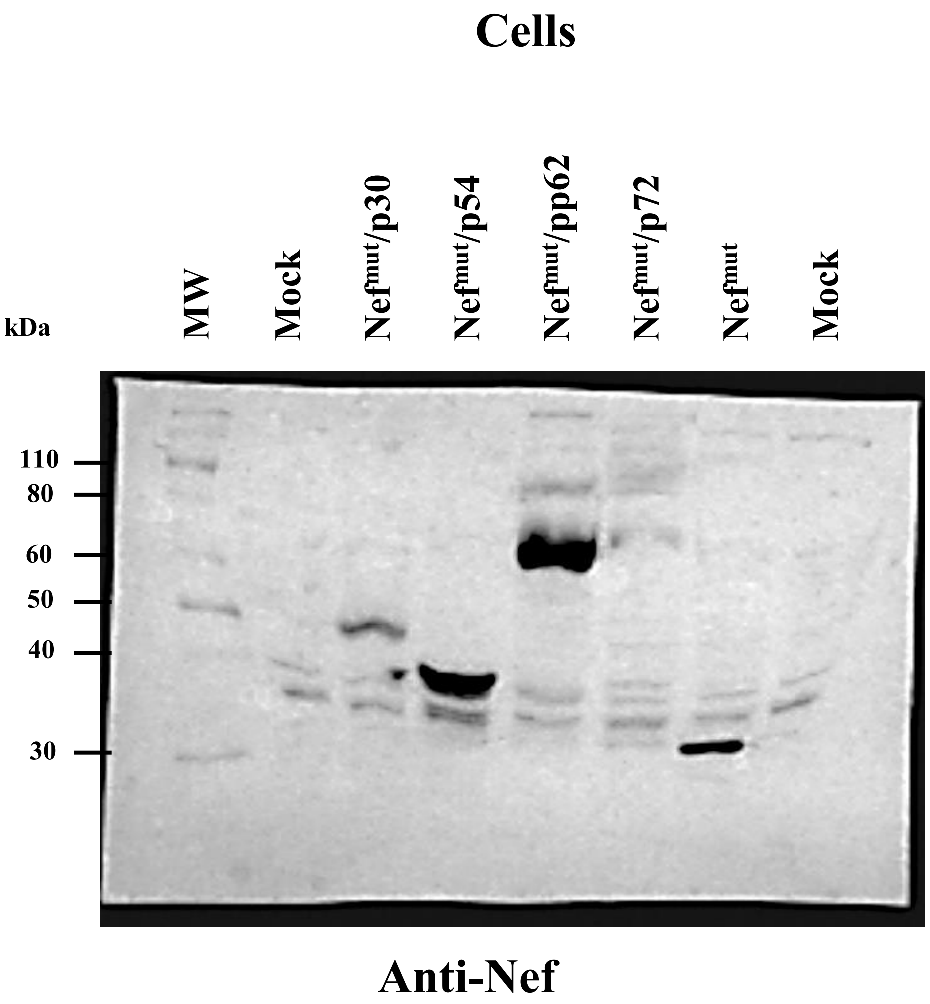

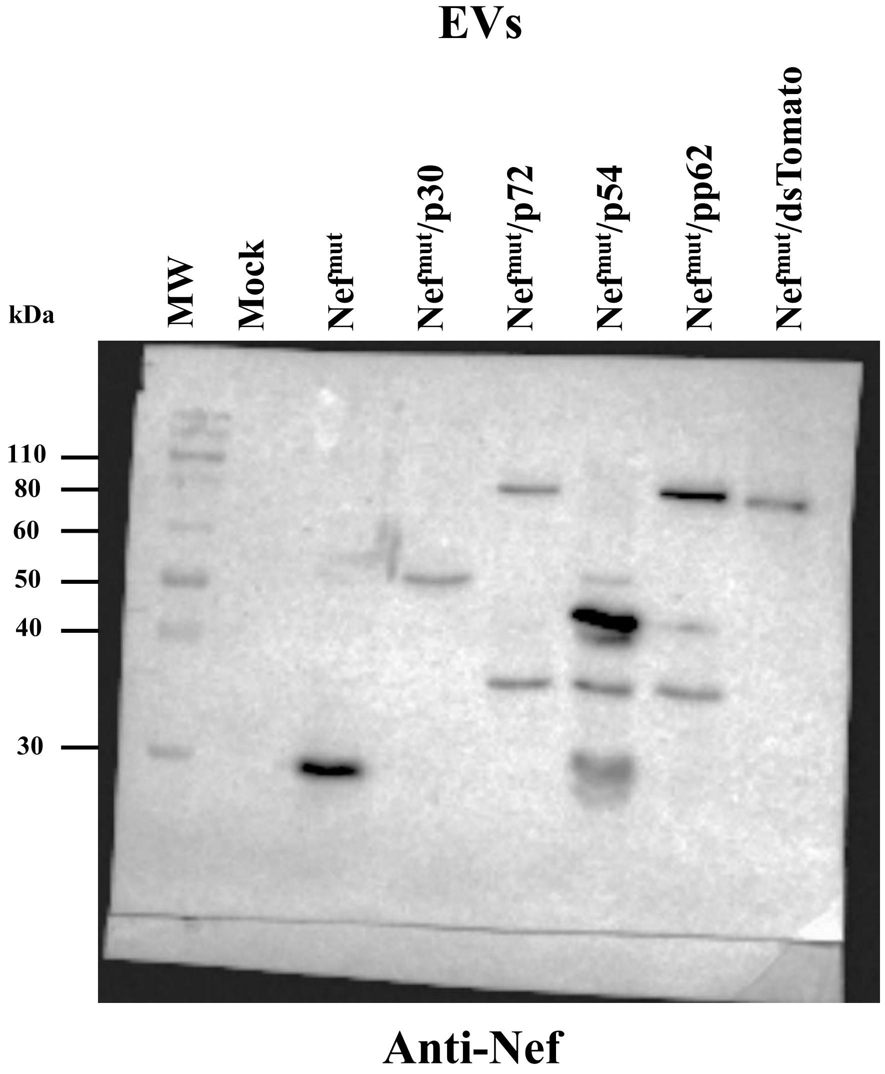

Supplementary figure S1. Western blot analysis. Raw data

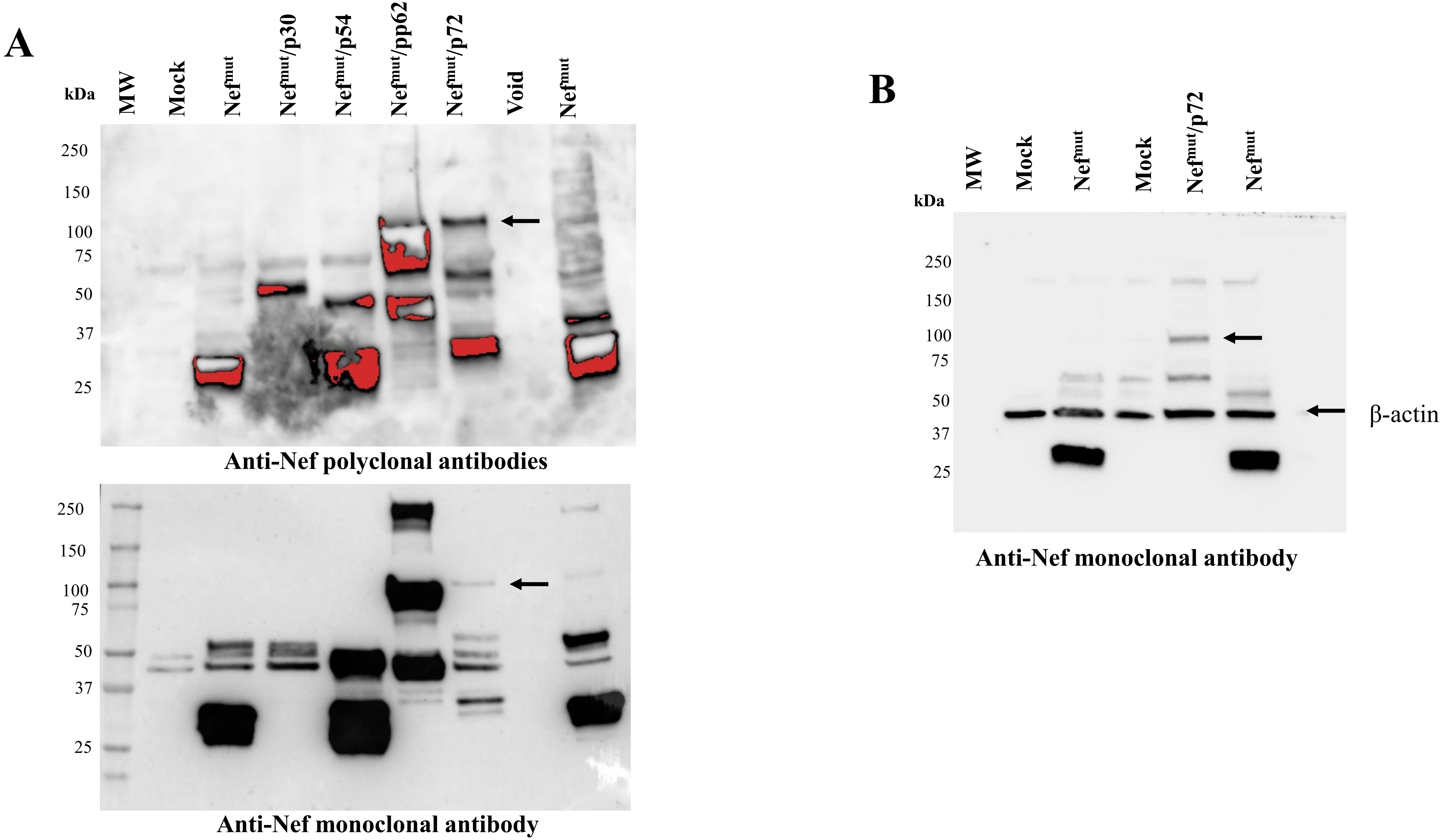

Supplementary figure S2. Western blot analysis on cell lysates from replicated transfection experiments with the indicated vectors. A. The whole panel of ASFV-related products was detected by both polyclonal and monoclonal anti-Nef antibodies. B. The analysis has been reproduced in cells expressing either Nef^{mut} or Nef^{mut}/p72 or left untreated (Mock), and the filter was incubated with both anti-Nef and anti-β-actin antibodies. Arrows indicate the signals of both Nef^{mut}/p72 and β-actin.

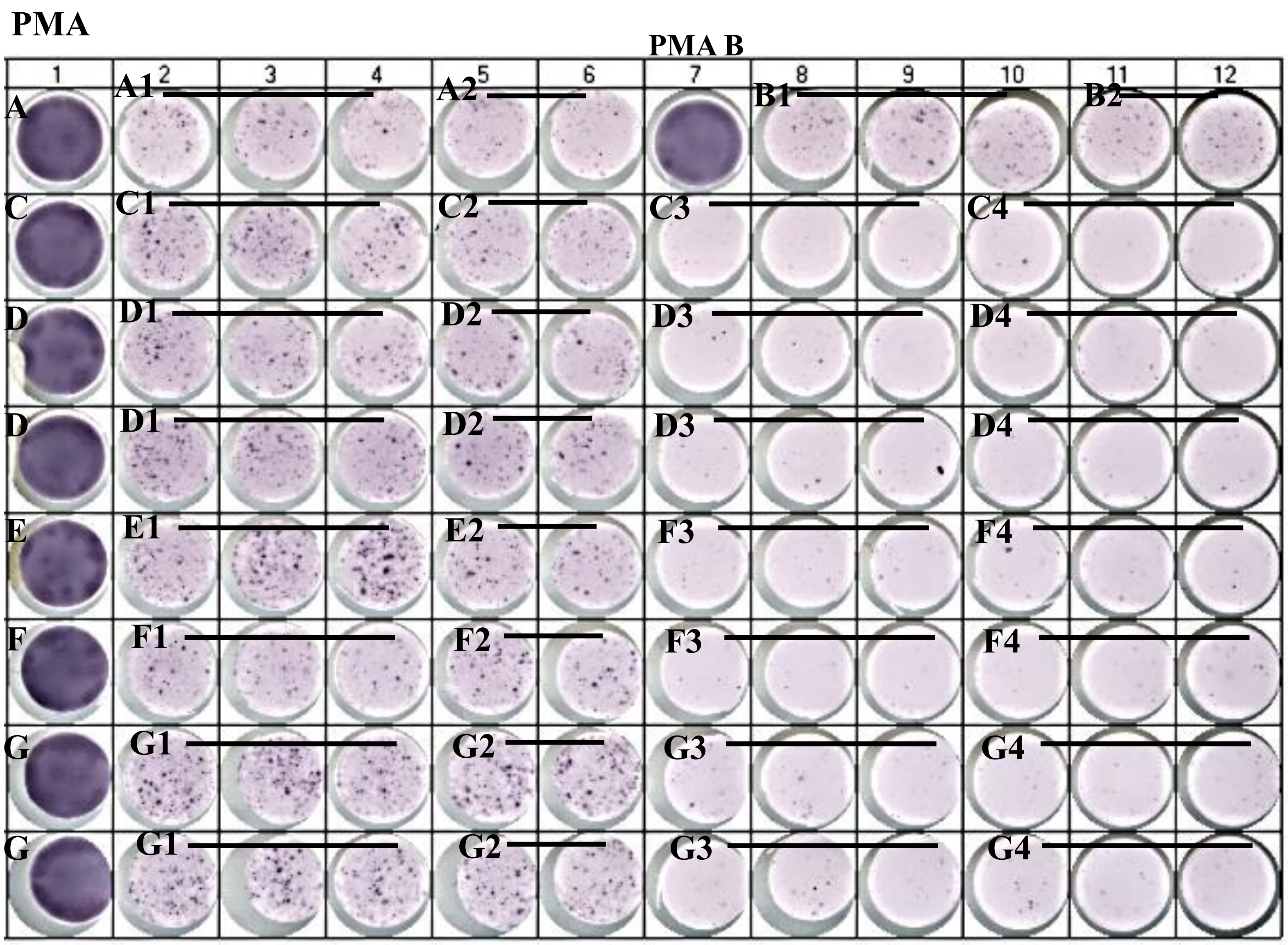

Challenging EVs:

A: nihil
B: mock EVs
C: Nef^{mut} EVs
D: Nef^{mut}/p30 EVs
E: Nef^{mut}/p54 EVs
F: Nef^{mut}/pp62 EVs
G: Nef^{mut}/p72 EVs

EliSpot conditions:

1: pools of specific peptides*
2: pool of Nef peptides
3: nihil
4: pool of unrelated peptides

*** In this assay, both A and B conditions were treated with the p72-specific pool of peptides**

Supplementary figure S3. Raw data from a representative IFN-γ EliSpot analysis carried out with cells recovered after priming assays**.** Conditions and treatments are indicated on the right. Bars indicate the well replicates.

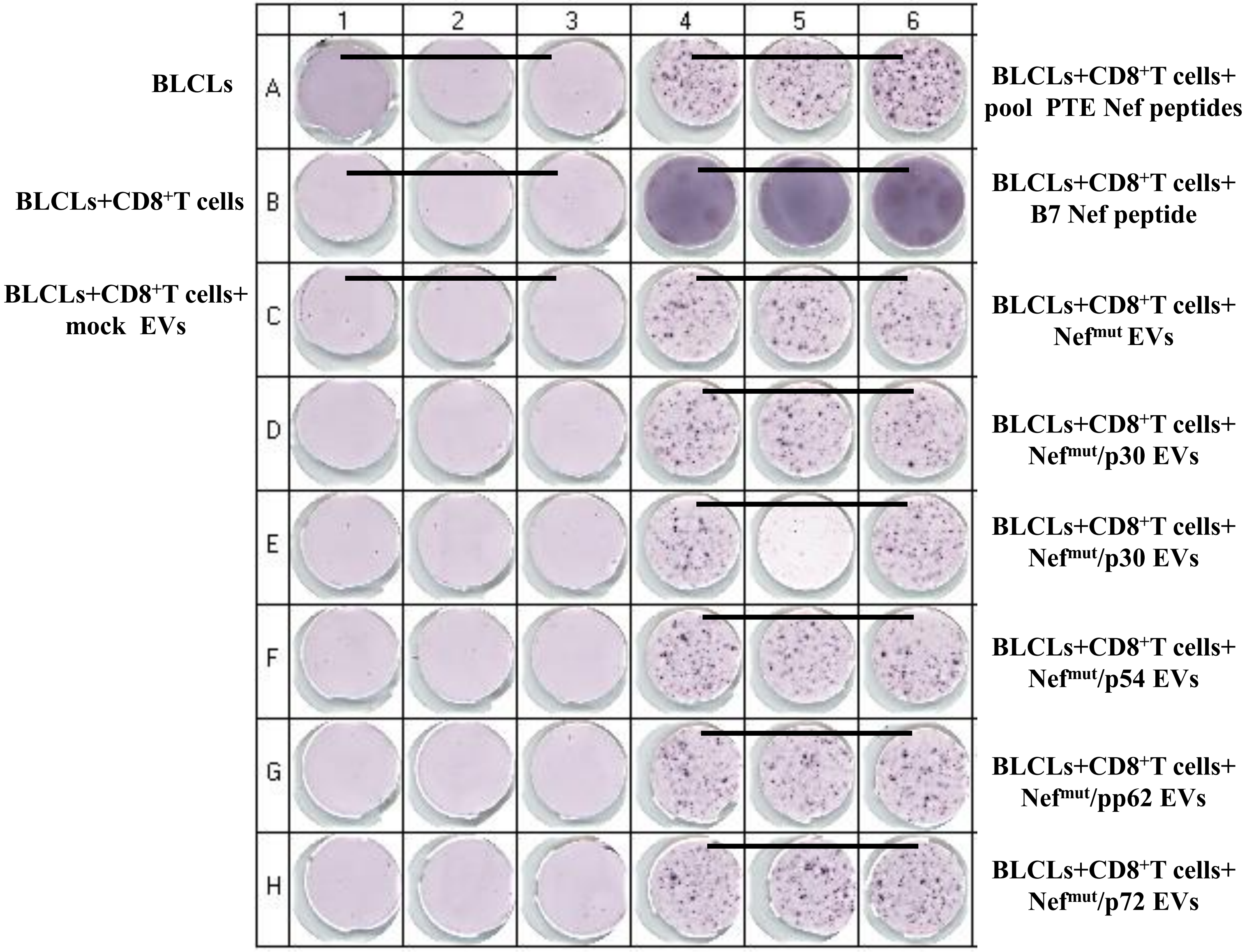

Supplementary figure S4. Raw data of IFN-γ EliSpot analysis in the context of cross-presentation assay**.** Conditions and treatments are indicated. Bars indicate the well replicates**.**
